# Immunomagnetic Sample Preparation Targeting Host- and Viral-Derived Antigens for HIV‑1 Isolation from Limited Plasma Volumes

**DOI:** 10.1021/acs.analchem.5c06176

**Published:** 2026-04-29

**Authors:** Gaurav K. Gulati, Nuttada Panpradist, Barry R. Lutz, James J. Lai

**Affiliations:** † Department of Bioengineering, 34878 University of Washington, Seattle, Washington 98195, United States; § Department of Materials Science and Engineering, National Taiwan University of Science and Technology, Taipei 106335, Taiwan

## Abstract

Rapid and accurate HIV detection is crucial for early diagnosis and viral load (VL) monitoring in individuals receiving antiretroviral therapy (ART). To address the challenge of limited sample volume, we developed a novel immunomagnetic sample preparation method to isolate HIV-1 virions from 25 μL of plasma—about one-tenth of a small finger-prick sample. Immunomagnetic conjugates were produced by coupling antimouse IgG magnetic beads with mouse IgG antibodies targeting host- and virus-derived antigens on the HIV surface. In buffer spiked with inactivated HIV-1 grown in the HUT-78 cell line, these conjugates achieved capture efficiencies of 45–97%. Optimization of conjugate concentrations (0.71–5.71 mg/mL) improved capture efficiency by 19%, with 15 min identified as the optimal capture time. In spiked plasma (25 μL), immunoconjugates targeting different surface antigens captured inactivated HIV-1 across inputs of 148–53,145 RNA copies/25 μL (5,920–2,125,800 RNA copies/mL) with consistent efficiencies ranging from 41% to 100%. In clinical specimens from individuals living with HIV with VLs ranging from 100,000 to >1 million copies/mL, capture efficiencies varied from 5% to 60% depending on the conjugate used. Notably, combining CD44 and CD46 targeting conjugates, which recognize different surface markers, did not enhance virion capture, suggesting the coexpression of targeted antigens on HIV-1. Together, our results established immunomagnetic isolation as a promising approach for HIV-1 sample preparation, enabling more accessible, sensitive, and rapid VL quantification.

## Graphical abstract

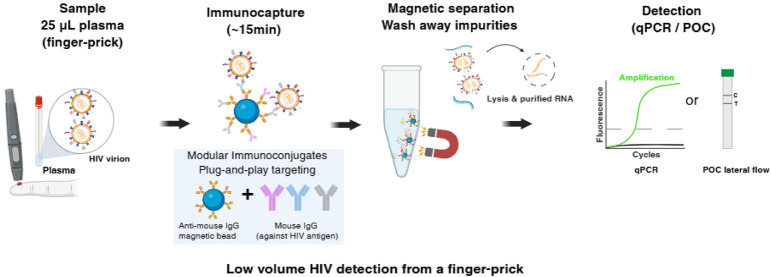

## Introduction

Approximately 39.9 million people worldwide were living with HIV (PLHIV) in 2023, with 14% unaware of their status.^1^ Early diagnosis during acute infection (<3 months postinfection), when HIV antibodies are absent and only HIV RNA or p24 antigen is detectable, is critical because individuals in this stage account for 10–50% of new transmissions due to their undiagnosed status and high viral loads (VLs).^2,3^ Routine VL monitoring is also essential for PLHIV on antiretroviral therapy (ART) to detect viral rebound and guide treatment adjustments.^3,4^ However, access to VL testing remains limited in many settings. In 2013, fewer than 20% of treated individuals in Africa had access to VL testing,^5^ and by 2016 only 13–37% in Africa and <50% globally were receiving routine VL monitoring.^6^ Centralized laboratory testing with multiday turnaround times further contributes to patient loss to follow-up; for example, in 2015, 25% of diagnosed individuals in Washington, DC and ∼33% in South Africa failed to connect to care due to delays.^7^ These barriers underscore the urgent need for rapid, accessible VL testing platforms.

While rapid and decentralized testing technologies have expanded access to HIV screening and monitoring, existing workflows still face significant analytical constraints when specimen volumes are limited.^2,3,8^ Fourth-generation rapid tests, such as the Alere Determine HIV-1/2 Ag/Ab Combo, detect p24 antigen, which becomes detectable roughly 2 weeks postinfection, but perform poorly in detection, with low specificity (0.983) observed in acute infection.^8^ These antigen/antibody tests are also unsuitable for monitoring viral rebound in ART-treated individuals. Nucleic acid amplification test (NAAT)-based devices, such as the GeneXpert HIV-1 viral load assay, integrate sample preparation, amplification, and detection to improve accessibility, but remain costly, require stable infrastructure, and typically demand ∼1 mL plasma, necessitating phlebotomy and centrifugation.^9,10^ Thus, assay sensitivity is often achieved at the expense of specimen volume and operational complexity, limiting accessibility in decentralized settings.

Efforts to adapt NAATs to smaller volumes (<100 μL) have also faced challenges. Low-volume samples inherently contain fewer RNA copies, and extraction efficiency decreases disproportionately as specimen volume is reduced, amplifying the impact of RNA loss during handling and purification.^4,10–12^ For example, Xpert HIV testing with 100 μL whole blood achieved a limit of detection (LoD) of ∼200–300 copies/mL,^13,14^ compared to ∼5 copies/mL when using 1 mL plasma.^15^ The LIAT Analyzer (IQuum) improved sensitivity by incorporating bead-based RNA enrichment, but still required 150–200 μL plasma or 75 μL whole blood to achieve a LoD of 57 copies/mL.^4,12^ These observations underscore that RNA extraction efficiency—not amplification chemistry alone—becomes the dominant constraint in microvolume viral load testing.

Comparative studies confirm that larger plasma inputs substantially improve detection. Using 25 μL plasma, the Amplicor assay achieved an LoD of ∼164 copies/mL, whereas increasing to 500 μL with QIAamp extraction reduced the LoD to ∼15 copies/mL.^16^ Similarly, increasing specimen volume from 25 to 250 μL lowered the LoD from 400 to 50 copies/mL.^17^ Although specialized formats have demonstrated feasibility with as little as 25–50 μL plasma,^18^ extraction efficiency generally decreases with smaller volumes due to both reduced target copies and greater loss during handling. Small-volume testing remains valuable for finger-prick and pediatric sampling, and methods that improve virion isolation and RNA recovery from small plasma volumes may help mitigate volume-dependent performance losses and support more reliable VL measurement.

Immunomagnetic virion capture offers a promising solution. This strategy, successfully applied to viruses such as SARS-CoV-2 and influenza, enhances RNA integrity, reduces inhibitors, and improves sensitivity in NAAT workflows.^19–22^ Importantly, targeting both viral surface antigens and host-derived antigens incorporated into the HIV envelope during budding can increase capture specificity and reduce reliance on extensive plasma processing.^23–26^

In this work, we define immunomagnetic virion capture as a modular, volume-preserving sample-processing step specifically tailored for microvolume HIV viral load testing (≤25 μL plasma), a regime in which conventional extraction-based workflows experience disproportionate RNA loss and reduced analytical sensitivity. Rather than introducing new bead chemistries or capture architectures, the principal novelty of this study lies in the systematic selection and validation of host-derived antigens incorporated into the HIV envelope as capture targets, and in establishing an analytically constrained operating framework for reliable virion recovery. By intentionally employing standardized, commercially available reagents and workflows, we isolate the analytical contribution of the capture step independent of materials innovation, enable direct comparison with existing small-volume workflows, and facilitate reproducibility and adoption across laboratories. Within this framework, we evaluate capture performance across clinically relevant viral load and specimen-volume regimes, thereby defining the conditions under which immunomagnetic capture can support sensitive and scalable low-volume HIV nucleic acid testing.

## Experimental Section

### Materials

MagnaBind Goat Anti-Mouse IgG Beads, Cat # 21354, Thermo Scientific; Alexa Fluor 488 ChromPure Mouse IgG, whole molecule, Cat # 015540-003, Jackson ImmunoResearch Europe LTD; HIV-1 culture fluid (heat-inactivated, HUT 78 cell line origin, 2.6 × 10^10^ copies/mL), Cat # 0801032CFHI; HIV RNA, Cat # VR-3351SD, ATCC; bovine serum albumin, Cat # A2153, Sigma; RNA UltraSense One-Step Quantitative RT-PCR System, Cat # 11732927, Thermo Scientific; Human plasma (50 mL), Innovative Research, Inc. Mouse IgG antibodies: CD8 (UCH-T4), Cat # sc-1181, Santa Cruz Biotechnology, Inc.; CD18 (Integrin beta-2), Clone ID H52-s, DSHB (Developmental Studies Hybridoma Bank); HIV1gp120, Cat # sc-57810, Santa Cruz Biotechnology, Inc.; CD26, Cat # 555435, BD Pharmingen; integrin alpha-4, Clone ID P4G9, DSHB; CD43 (DF-T1), Cat # sc-6256, Santa Cruz Biotechnology, Inc.; CD46 (1E3D1), Cat # MA5-29113, Invitrogen; β_2_-microglobulin (B2M-01), Cat # MA1–19141, Invitrogen; integrin alpha-L (CD11a), Clone ID MHM.24, DSHB; CD86 (B7-2), Clone ID IT2.2, Invitrogen; CD44, Clone ID H4C4, DSHB; ICAM1, Clone ID P2A4, DSHB; CD45, Clone ID H5A5, DSHB; HLA-DR, Clone ID LN3, Cat # 14-9956-82, Thermo Fisher Scientific; Tim-4 (human):Fc (mouse) (rec.), Cat # CHI-HF-211T4-C100, AdipoGen Life Sciences. IgG1, K isotype, Cat # 555746, BD Pharmingen.

### Immunomagnetic Conjugate Preparation

The conjugates were prepared by immobilizing mouse IgG onto antimouse IgG magnetic beads following a modified version of the manufacturer’s protocol (MagnaBind, Thermo Scientific). MagnaBind Goat Anti-Mouse IgG are superparamagnetic particles (1–4 μm diameter) used for antibody immobilization and immunocapture-based separation assays.^27,28^ These beads enable efficient antibody immobilization and rapid magnetic separation, supporting efficient virion isolation from small-volume plasma in this study. Briefly, 1 mg of magnetic beads (1 mg/mL, 10^8^ beads/mg) was washed three times with 1 mL of PBS buffer (pH 7.4) using magnetic separation. The washed beads were then resuspended in 250 μL of PBS containing 0.1% BSA, resulting in a final bead concentration of 1 mg/250 μL. A 200 μL aliquot of the bead suspension (0.8 mg beads) was incubated with 1–8 μg of mouse IgG, bringing the total reaction volume to 300 μL. The mixture was gently mixed on a rotor for 20 min at room temperature. After incubation, the beads were washed three times with 500 μL of sterile PBS containing 0.1% BSA via magnetic separation. The final conjugates were resuspended in 139 μL of sterile PBS with 0.1% BSA. For Tim-4 conjugation, TBS or TBS with 0.1% BSA was used instead of PBS.

### Extraction and Quantification of Viral RNA Using RT-qPCR

Viral RNA was extracted and purified from magnetic bead-bound virions using the Qiagen Viral RNA Extraction and Purification Kit, following the manufacturer’s protocol with slight modifications. Magnetic beads bound to virions were separated from HIV-positive plasma or spiked buffer samples, washed twice, and reconstituted in PBS containing 0.1% BSA. The mixture was then combined with lysis buffer containing carrier RNA in a 1:4 ratio, vortexed, and incubated at room temperature (15–25 °C) for 10 min to ensure complete lysis. The beads were then removed via magnetic isolation. An equal volume of 96–100% ethanol was added to the lysed sample, which was vortexed, applied to a QIAamp Mini column, and centrifuged at 6,000 × *g* for 1 min. The flow-through was discarded, and the column was retained for further washing. The bound RNA was washed twice with Washing Buffer-1 (Qiagen) and twice with Washing Buffer-2. After the final wash, 60 μL of pre-equilibrated Elution Buffer (Qiagen) was added directly onto the column membrane to elute the purified RNA. The eluted RNA was stored at −80 °C until further analysis. Purified RNA was quantified using an in-house RT-qPCR assay targeting a conserved region of the HIV-1 LTR gene. RNA copy numbers were determined by comparison to a standard curve generated from known RNA concentrations (ATCC HIV-1 RNA), ensuring accurate and precise quantification.

### Immunocapture of Inactivated HIV-1 Virions

Inactivated HIV-1 virions (HIV-1 IIIB strain, derived from the HUT-78 cell line) were spiked into PBS containing 0.1% BSA at a concentration range of approximately 10^2^ to 10^6^ RNA copies. The immunocapture assay was performed in 2 mL Eppendorf tubes by mixing 0.8 mg of immunomagnetic conjugated beads with 140 μL of the spiked buffer specimen. The mixture was incubated at room temperature for 1 h with gentle mixing on a rotor unless otherwise specified. Following incubation, the beads were washed three times with 300 μL of PBS containing 0.1% BSA using magnetic separation to remove unbound virions. The beads were then resuspended in 140 μL of PBS containing 0.1% BSA, and RNA extraction was performed using a viral RNA extraction kit (RNA UltraSense One-Step Quantitative RT-PCR System, Thermo Scientific) following the manufacturer’s protocol. RNA quantification was conducted using an in-house RT-qPCR assay targeting a conserved region of the HIV-1 LTR gene. Capture efficiency (%) was calculated as the proportion of captured HIV RNA copies relative to the input RNA copies, with comparisons made between immunomagnetic conjugates and control beads (unconjugated beads or isotype controls). Nonspecific capture by control beads was also measured, and specific capture efficiency was determined after subtracting nonspecific RNA capture values. To assess the effect of conjugate quantity on capture efficiency, varying amounts of HLA-DR immunomagnetic conjugates (0.1, 0.2, and 0.8 mg) were incubated with inactivated HIV-1. For Tim-4 conjugate, TBS or TBS containing 0.1% BSA was used instead of PBS.

### Evaluation of Incubation Time for Maximum Capture Efficiency

Optimization experiments were conducted using human plasma spiked with inactivated HIV-1 (∼100,000 RNA copies/μL). Each isolation experiment was performed with 25 μL of spiked plasma, and 115 μL of PBS containing 0.1% BSA and 0.8 mg of anti-HLA-DR conjugates. The mixtures were incubated at room temperature for 15, 30, or 60 min on a rotor. Following incubation, the beads were washed three times with PBS containing 0.1% BSA using magnetic separation to remove unbound virions. RNA extraction and quantification were performed as previously described. Control beads (unconjugated or isotype) were processed in parallel for comparison. Capture efficiency (%) was calculated for each time point as the proportion of captured HIV RNA copies relative to the input RNA copies.

### Evaluation of Capture Efficiency across Viral Load Variations

The impact of specimen VL for the capture efficiency was evaluated using human plasma spiked with inactivated HIV-1. Low VL specimens contained 148–401 RNA copies/25 μL and high VL specimens contain 5221–53,145 RNA copies/25 μL. The virion isolation was performed as previously described (incubation time optimization) with 1 h incubation time. RNA extraction and quantification were performed using the Qiagen Viral RNA Extraction and Purification Kit. The evaluation utilized conjugates targeting HLA-DR, CD44, β2-microglobulin, CD26, and Tim-4-mFc. Control beads (unconjugated or isotype) were processed in parallel for comparison. Capture efficiency (%) was calculated for each time point as the proportion of captured HIV RNA copies relative to the input RNA copies. For Tim-4 conjugate, TBS or TBS containing 0.1% BSA was used instead of PBS.

### Evaluation of Capture Efficiency in HIV-Positive Plasma Samples

To evaluate the performance of immunomagnetic conjugates in clinical samples, HIV-positive plasma specimens with variable VLs were obtained from the Center for AIDS Research (CFAR) at the University of Washington (Seattle, WA). The VLs for Patient IDs #1236, 2048, 1654, 1087, 1266, and 1364 were 107024, 160584, 383964, 1393440, 126000, and 186048 copies/mL, respectively, as measured using our in-house RT-qPCR. Each virion isolation experiment was performed with 25 μL of plasma, 115 μL of PBS containing 0.1% BSA, and 0.8 mg of conjugates targeting various antigens. Virion isolation was conducted as previously described (incubation time optimization), with an incubation time of 1 h. RNA extraction and quantification were performed as previously described. Capture efficiency (%) was calculated as the proportion of captured HIV RNA copies relative to the input RNA copies. Control beads were processed in parallel to measure nonspecific capture.

### Evaluation of Capture Efficiency across Varying Plasma Volumes

To assess the consistency of capture efficiency across varying input viral loads (VLs), HIV-positive plasma (ID #1654, VL ∼ 384,000 copies/mL) was diluted in PBS to a final volume of 140 μL, using 2–50 μL of plasma per sample. This corresponded to input RNA copies ranging from 1,295 to 14,268, as measured by in-house RT-qPCR. Conjugates targeting CD46 (0.8 mg) were incubated with the samples for 1 h at room temperature. Virion isolation, RNA extraction, and quantification were performed as previously described. Capture efficiency (%) was calculated for each plasma volume, and Spearman’s correlation analysis was conducted to evaluate the relationship between input VL and capture efficiency.

### Evaluation of Conjugate Combinations for HIV-1 Capture

The evaluation was conducted using HIV-positive plasma (ID #1654, VL ∼ 384,000 copies/mL). Each experiment involved mixing a 25 μL aliquot of plasma with 115 μL of PBS containing 0.1% BSA and 0.8 mg of conjugates. After a 1-h incubation at room temperature, virion isolation, RNA extraction, and quantification were performed as previously described. Capture efficiency (%) was calculated as the proportion of captured HIV RNA copies relative to the input RNA copies. Five experimental groups were tested: a no-capture control (no conjugate), an isotype control (conjugates with nonspecific mouse IgG), conjugates targeting CD44, conjugates targeting CD46, and conjugates targeting both CD44 and CD46. For the combination group, CD44 and CD46 conjugates were added in equal amounts (0.8 mg each).

### Statistical Analysis

All experiments were performed in at least three independent replicates. Statistical comparisons between two groups were performed using the nonparametric Mann–Whitney U test, and comparisons among more than two groups were performed using the Kruskal–Wallis test. A *p*-value of <0.05 was considered statistically significant.

## Results

### Immunomagnetic Conjugates Capture Inactivated HIV Spiked into Buffer and Healthy Human Plasma

To selectively target and capture HIV-1, we developed multiple immunomagnetic conjugates by coupling antimouse IgG-coated magnetic beads with mouse IgG antibodies that specifically recognize viral surface markers or host antigens present on HIV-1 virions. Table 1 provides a literature review of the targeted antigens on the HIV envelope, categorized by the cell types from which HIV acquires them and the stages of infection during which they are predominantly expressed.^29–43^

**Table 1:** An Overview of the Targeted Antigens on the HIV Envelope^a^

Targeted viral or host-derived protein	Predominant host-cells involved^b^	Relative availability of targeted protein on HIV based on stage of infection^c^	References
gp120		Acute HIV infection	^30^
gp41		Acute HIV infection	^30^
CD26	T-lymphocytes and monocytes	HIV infection	^31^
CD43	T-lymphocytes	HIV infection	^32^
CD44	T-lymphocytes, macrophages and dendritic cells	Acute = chronic HIV infection	^34^
CD45	T-lymphocytes and monocytes	HIV infection	^35^
CD46	Dendritic cells	HIV infection	^36^
CD86	Macrophages and dendritic cells	HIV infection	^37^
CD54 (ICAM-1)	T-lymphocytes, macrophages and dendritic cells	HIV infection	^41^
CD11a (LFA-1)	T-lymphocytes, monocytes and macrophages	HIV infection	^31,38,41^
Integrin α4	Gut associated T-lymphocytes	Acute infection > Chronic infection	^40^
β_2_-microgloubin	Nucleated cells	HIV infection	^42^
Phosphatidylserine^d^	Abundantly present in plasma membrane of host cell	HIV infection	^43^
HLA-DR	Dendritic cells and macrophages	Chronic infection	

a These antigens are categorized by the cell types contributing to their presence and the infection stage (acute or chronic) during which they predominantly express on HIV.

b Immune cells are the primary cells involved in HIV-1 infection. During the eclipse phase (∼10 days post infection), primary founder virus could infect CD4+ T cells with greater efficiency than they could infect monocytes and macrophages (McMichael et al. 2010).

c Acute (<100 days post infection) and chronic (>100 days post infection).^100^

d Phosphatidylserine in the viral envelop binds with TIM4 protein highly expressed by macrophages.

To determine the mouse IgG binding capacity for the magnetic beads, Alexa Fluor-labeled mouse IgG was used as the model analyte. Our results indicate a binding capacity of approximately 1 μg of mouse IgG per 0.8 mg of magnetic beads in a 1 mL buffer system (Figure S1). We postulate that this binding capacity remains consistent for all mouse IgG targeting host or viral antigens when using the same standardized immobilization protocol. Theoretically, 1 μg/mL of an IgG antibody (∼6.67 nM), assuming a dissociation constant (*K*_D_) of 6.67 nM, could recognize ≥50% of the target antigen when the antigen concentration is below 0.1 nM (∼10^13^ molecules/L). Given the estimated surface antigen density of ∼30 gp120 molecules per virion,^44–47^ the equilibrium binding model indicates that 1 μg/mL of IgG antibody (0.8 mg/mL beads) can bind ≥50% of surface antigens when the VL is ≤10^9^ virions/mL. While these calculations are based on specific assumptions, they provide a reasonable estimate of the antibody quantity required for efficient HIV capture in this study.

Immunomagnetic conjugates were evaluated by measuring the percentage of HIV-1 virion captured and recovered from 140 μL virion-spiked buffer or plasma as quantified by RT-qPCR (Figure 1A, described in Capture of Inactivated HIV-1 in Buffer within Materials and Methods). When inactivated HIV-1 grown in the HUT-78 cell line was spiked into a buffer, conjugates targeting HLA-DR, Tim-4:Fc (human Tim-4 and mouse Fc), CD44, β_2_-microglobulin, and CD26 exhibited antigen-specific virion capture, with mean capture efficiencies ranging from 45 to 97% (Figure 1B). In contrast, the bead-only and isotype controls exhibited less than 30% nonspecific capture. The order of capture efficiency based on surface antigen targeting was: HLA-DR > Tim-4:Fc > β_2_-microglobulin > CD44 > CD26. Conjugates targeting gp120, gp41, CD18, CD11a, CD43, CD46, CD8, CD54, CD45, and integrin α4—showed capture efficiencies similar to or lower than the nonspecific background capture observed with controls. This suggests that these markers are either minimally present or absent on inactivated HIV-1 virions grown in the HUT-78 cell line.

**Figure 1:**
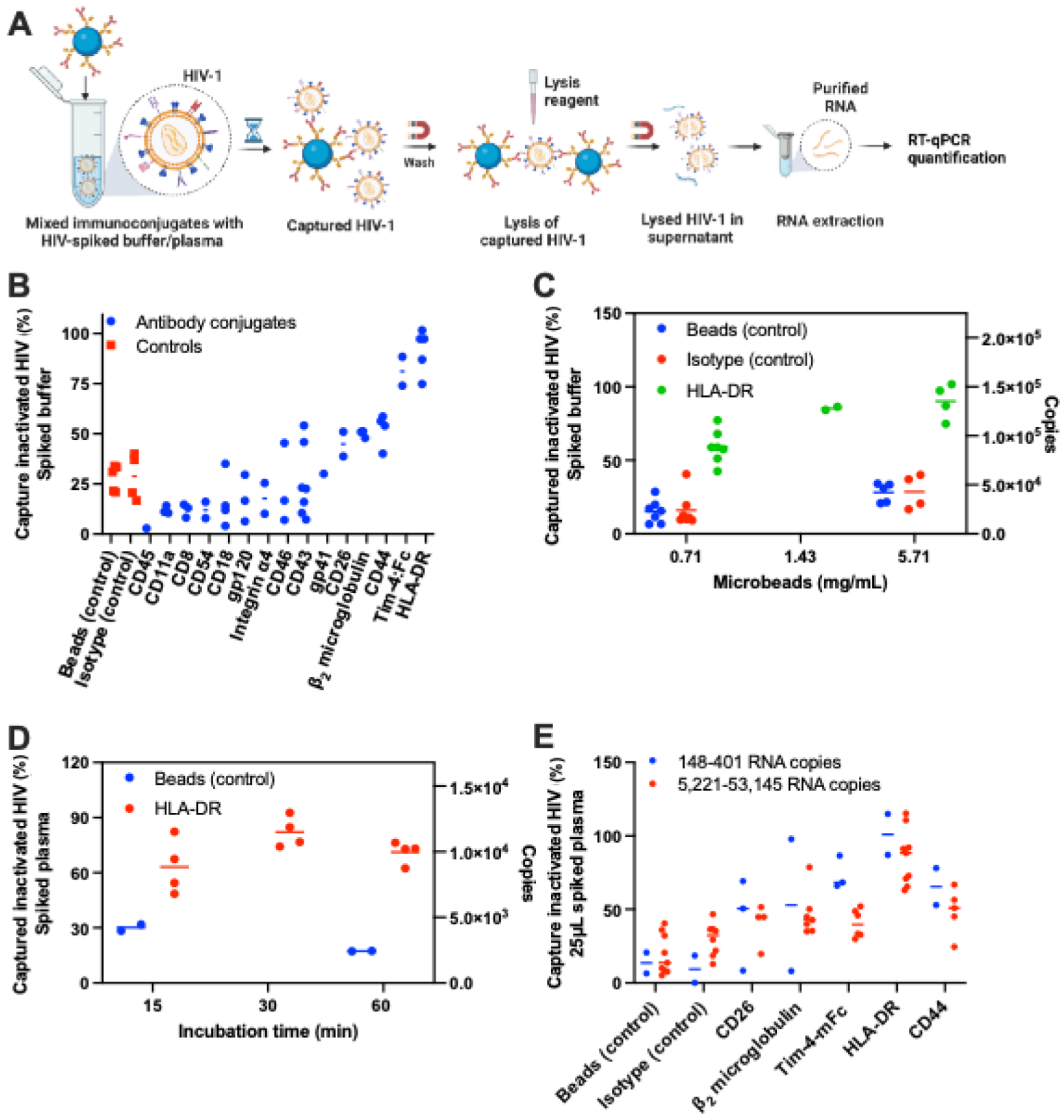
Immunomagnetic conjugate-mediated capture of inactivated HIV-1 spiked into buffer or healthy human plasma. A. The workflow illustrates the process of HIV-1 virion capture using immunomagnetic conjugates. Inactivated HIV-1 was spiked into 140 μL of buffer or into 25 μL of healthy human plasma diluted to 140 μL with buffer containing 0.8 mg of immunomagnetic conjugate or control microbeads and incubated for 1 h, unless otherwise indicated. The virions bound to the immunoconjugates were then magnetically isolated and washed. The HIV RNA was extracted, purified, and quantified by RT-qPCR to determine the captured inactivated HIV-1 (%). B. % Captured inactivated HIV-1 by various immunomagnetic conjugates or controls when incubated with buffer spiked with inactivated virions at 15,000–344,000 RNA copies. C. % Captured inactivated HIV-1 using different quantities of anti-HLA-DR immunoconjugates or control microbeads ranging from 0.71–5.71 mg/mL when incubated with buffer spiked with virions at 150,000 RNA copies. D. % Captured inactivated HIV-1 by anti-HLA-DR immunoconjugates or controls when incubated for different times in diluted healthy human plasma, 25 μL, spiked with inactivated HIV-1 at 14,000 RNA copies. E. % captured inactivated HIV-1 by selected immunoconjugates when incubated with human plasma spiked with inactivated virions at low (148–401) and high (5221–53145) RNA copies.

We selected the anti-HLA-DR conjugate (the highest capture efficiency in Figure 1B) to assess how capture efficiency varies with the conjugate quantity (Figure 1C). Increasing the conjugate quantity from 0.1 mg (0.71 mg/mL) to 0.2 mg (1.43 mg/mL) and 0.8 mg (5.71 mg/mL) improved mean capture efficiency from 59% to 90%. Similarly, increasing the amount of bead-only and isotype controls, corresponding to the same concentrations, resulted in a proportional increase in nonspecific capture efficiency (15–29%). After subtracting the nonspecific capture observed with the isotype control, the estimated specific capture by the HLA-DR immunoconjugate ranged from 43–62%, representing a net increase in capture efficiency of nearly 20% when the conjugate quantity was increased from 0.71–5.71 mg/mL. The average capture efficiency with 5.71 mg/mL of anti-HLA-DR conjugate achieves maximum virion capture, isolating up to 90% of virions from 140 μL of buffer spiked with 150,000 HIV-1 RNA copies.

To determine the optimal capture time in a biologically relevant matrix, healthy human plasma, 25 μL, was spiked with inactivated HIV-1 (∼14,000 RNA copies) and incubated with anti-HLA-DR conjugates at 5.71 mg/mL or with control beads for 15 to 60 min. Figure 1D shows the capture efficiency of anti-HLA-DR conjugates and bead-only controls at various time points. At all tested incubation times, the conjugates exhibited significantly higher (*p* < 0.05) capture efficiency (ranging from 63 to 82%) compared to bead-only controls (15 to 30%). However, no significant difference (*p* > 0.05) was observed when comparing conjugate capture efficiency at 15, 30, and 60 min, suggesting that a minimum capture time of 15 min is sufficient for effective HIV-1 capture. To ensure consistency across experiments, a 1-h capture time was used for all subsequent experiments unless otherwise specified.

To evaluate the immunoconjugates in capturing virions from a small specimen volume at different VL levels, we spiked inactivated HIV-1 into 25 μL of healthy human plasma to create low VL samples (148–401 RNA copies) and high VL samples (5,221–53,145 RNA copies). Conjugates targeting HLA-DR, CD44, β_2_-microglobulin, CD26, and Tim-4:Fc were tested against these spiked plasma samples based on their demonstrated success in spiked buffer (Figure 1B). Figure 1E shows the capture efficiency of bead-only controls and immunomagnetic conjugates for HIV-1 spiked in plasma. Bead and isotype controls exhibited a mean capture efficiency of 9–14% in low VL samples and 19–30% in high VL samples, indicating nonspecific capture. For both low- and high-VL samples, the order of capture efficiency across conjugates was the same. For HLA-DR, CD44, β2-microglobulin, CD26, and Tim-4:Fc, the mean efficiencies were 100, 65, 53, 43, and 74% in low-VL samples and 86, 49, 47, 40, and 41% in high-VL samples, respectively. No significant difference was observed in mean capture efficiency between low and high VL samples for each conjugate, indicating consistent performance across VLs.

### Immunomagnetic Conjugates Capture HIV-1 from Clinical Specimens

To evaluate the efficiency of capturing virions from clinical specimens, the conjugates were incubated with 25 μL of HIV-positive plasma. Plasma specimens (*n* = 3–5) with VLs ranging from 107,024 to 1,393,440 RNA copies/mL were tested. The average capture efficiencies for different surface antigen targets are summarized in Figure 2A as follows: CD54 (5%), CD11a (5%), integrin α4 (16%), β_2_-microglobulin (14%), Tim-4:Fc (18%), HLA-DR (28%), CD43 (29%), CD46 (37%), and CD44 (50%). Notably, conjugates targeting HLA-DR, CD43, CD46, and CD44 exhibited significantly higher mean capture efficiencies (*p* < 0.05) compared to the nonspecific capture observed with the isotype or bead controls (3–4%). In contrast, conjugates targeting gp120, gp41, CD26, CD45, and CD86 showed capture efficiencies comparable to the nonspecific background observed with the controls. Healthy plasma, used as a negative control, showed no detectable HIV RNA. To further assess the reproducibility of capture performance across clinical specimens, variability metrics were calculated for the four most effective targets. Across patient plasma samples, mean capture efficiencies and associated variability were as follows: HLA-DR (average 28%, CV 31%, 95% CI 22–33%), CD43 (average 29%, CV 24%, 95% CI 23–34%), CD46 (average 37%, CV 36%, 95% CI 27–47%), and CD44 (average 50%, CV 30%, 95% CI 41–58%). These results demonstrate antigen-specific capture of HIV-1 from clinical plasma from PLHIV, using immunomagnetic conjugates, particularly those targeting HLA-DR, CD43, CD46, and CD44 antigens.

**Figure 2:**
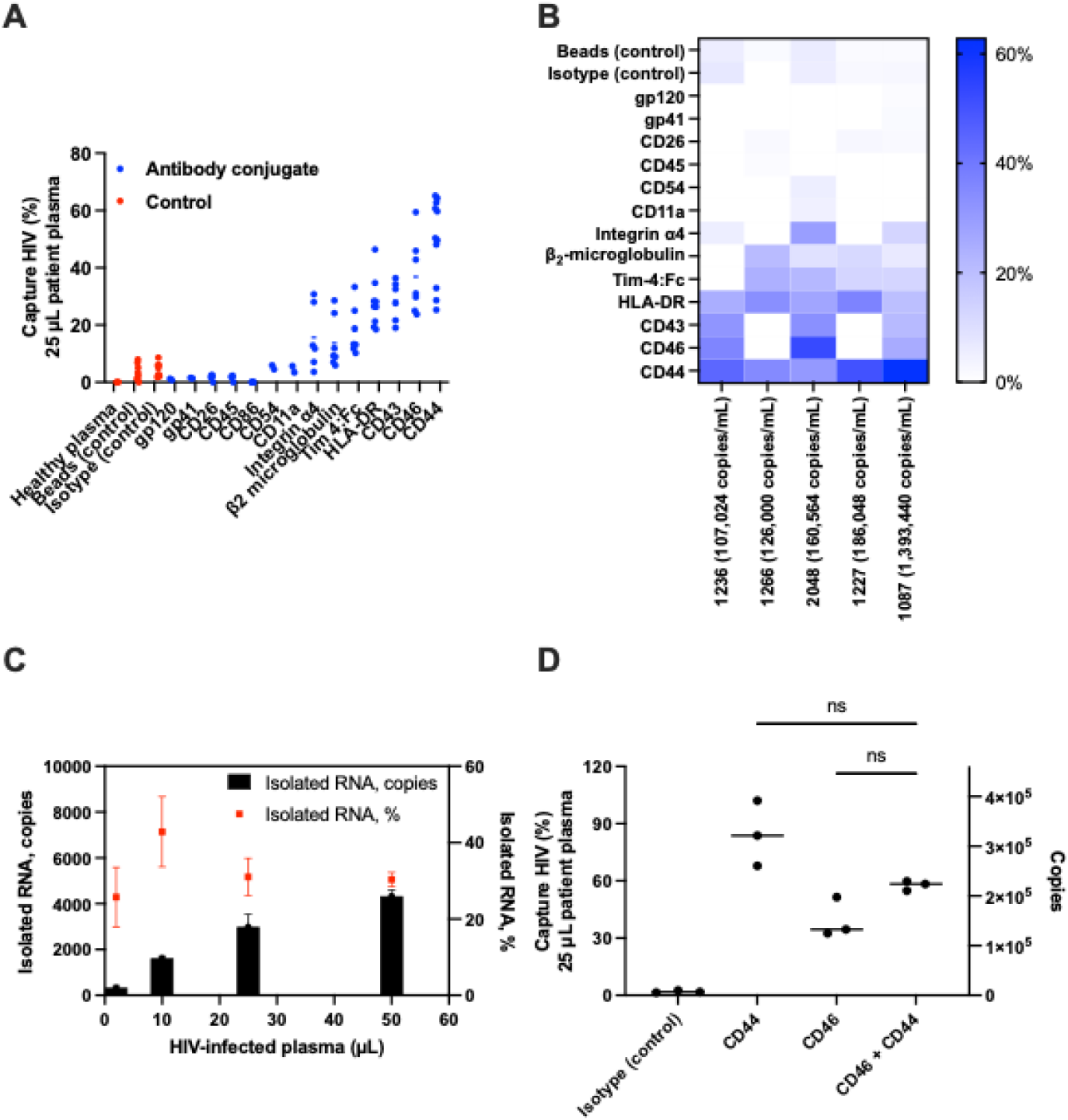
Immunoconjugate-mediated capture of HIV-1 from clinical specimens. Five specimens with viral loads ranging from 107,024 to 1,393,440 copies/mL were used. *n* = 2–3 technical replicates/specimen and *n* = 3–5 clinical specimens/conjugate, except gp120, gp41, CD45, CD86, ICAM-1, and CD11a/CD18 LFA-1 (*n* = 1 specimen) were used. A. % Captured HIV-1 by immunoconjugates incubated with HIV-positive plasma. Healthy human plasma acted as a negative control. Beads or isotype controls show nonspecific capture of HIV-1. B. Captured HIV-1 RNA copies by immunoconjugates incubated with 25 μL patient specimens. C. Captured HIV-1 RNA copies by CD46 immunoconjugate incubated with increasing volumes of HIV-positive plasma (ID #1654, VL: 384,000 copies/mL), ranging from 2 to 50 μL (*n* = 2–3 technical replicates). D. % Captured HIV-1 by the combination of CD46 and CD44 immunoconjugate incubated with patient plasma (ID #1654, VL: 384,000 copies/mL). CD44 (1.6 mg) and CD46 (1.6 mg) were used alone or in combination (0.8 mg each, total 1.6 mg). The no capture control shows the input copies present in 25 μL of HIV-positive human plasma, measured by RT-qPCR. The isotype control shows nonspecific capture by IgG isotype armed microbeads. No-capture control served as the baseline HIV RNA copies in the different plasma volumes (*n* = 1–3 replicates).

To investigate the effect of VL on conjugate capture efficiency, patient specimens were categorized into low VL (100,000–200,000 copies/mL; 4 specimens, 2–6 replicates/specimen) and high VL (>1,000,000 copies/mL; 1 specimen, 2 replicates/specimen). In the low VL group (IDs #1236, 1266, 2048, and 1227), controls (isotype and beads) captured <1–7.2% (Figure 2B). In the high VL group (ID #1087), the nonspecific capture background was <2.5%. In the low VL group, the average capture efficiency per 25 μL plasma ranged from 13–50% for conjugates targeting integrin α4, β_2_-microglobulin, Tim-4:Fc, HLA-DR, CD43, CD46, and CD44, respectively. In the high VL group, the average capture efficiency ranged from 13–63%, respectively (Figure 2B). Consistent with these findings, analysis of CD44-targeted capture across four independent HIV-positive patient specimens with VLs between 107,000 and 186,000 copies/mL yielded a mean capture efficiency of 42%, with a CV of 34% and a 95% CI of 32–52%. These results confirm prior findings in contrived samples (Figure 1E), demonstrating that the capture efficiency of immunoconjugate remains consistent across specimens with variable VLs.

To further evaluate the consistency of virion isolation, we incubated CD46 conjugates with a patient specimen (VL = ∼384,000 copies/mL) at sample volumes of 2, 10, 25, and 50 μL (3 replicates/volume), resulting in an increase in input HIV-1 RNA copies from 1,295 to 14,268 (Figure 2C). The mean number of isolated HIV RNA copies increased from 333–4,328 for plasma volumes of 2 to 50 μL, respectively. The mean capture efficiency ranged from 26–42% relative to the total RNA input measured by the standard extraction (no immunocapture step) method on the same specimen. This range corresponds to approximately 2-fold variability in RNA recovery across input volumes and a 58–74% loss in RNA signal attributable to the immunocapture process under the specific conditions evaluated here. Importantly, no significant correlation was observed between mean capture efficiency (%) and input HIV copies.

To further improve virion capture efficiency, we investigated the potential of combining CD46 and CD44 targeting conjugates. We hypothesized that since the conjugates target distinct antigens on virions, their combination could result in increased capture efficiency. Figure 2D displays the HIV RNA copies captured by individual and combined conjugates. Each experiment utilized 25 μL of plasma from PLHIV, containing 9,624 HIV RNA copies. The isotype control group resulted in <2% nonspecific capture. Consistent with prior results (Figure 2B), the anti-CD44 and anti-CD46 conjugates resulted in 85% and 40% average capture efficiency (Figure 2D). However, combining anti-CD44 and anti-CD46 conjugates resulted in nearly 60% average capture efficiency, which was not significantly different than the anti-CD44 or anti-CD46 conjugate alone (*p* > 0.05). This suggests that these antigens are coexpressed on the same virions, providing no additional benefit when combined.

## Discussion

In this study, we introduce a novel sample preparation approach utilizing immunomagnetic conjugates that specifically target both viral surface antigens and host cell–derived antigens incorporated into the viral envelope during viral budding. The method is designed to operate under the strict copy-number and specimen-volume constraints of microvolume HIV testing and serves as a modular upstream processing step rather than a complete diagnostic assay. The approach utilizes a 25 μL plasma sample, making it compatible with fingerstick blood sampling. A rapid 15 min incubation of conjugates with virions in plasma at room temperature, followed by magnetic isolation, ensures efficient and clean sample preparation, suitable for subsequent amplification processes. Bead concentration and incubation time were optimized within defined analytical constraints, including limited antibody density per bead, small plasma input volumes (25 μL), clinically relevant viral load ranges, and biological variability among patient specimens. These conditions impose strict mass-balance limitations, where even modest virion losses can substantially affect analytical sensitivity. Within this constrained regime, the optimized parameters produce reproducible virion capture across antigen targets, viral loads, and specimen sources. By selectively isolating virions through host- and virus-derived surface markers, this approach enhances detection specificity. Through systematic, head-to-head evaluation of host- and virus-derived antigens, we identify HLA-DR, CD46, and CD44 as the most effective capture targets, demonstrating reproducible performance across a broad clinical viral load range (10^3^–10^6^ copies/mL). Importantly, the observed analytical sensitivity of approximately 148 copies per 25 μL plasma (5,920 copies/mL) supports the feasibility of this approach for low-volume viral load assays, where copy-number limitations are most restrictive.

Immunomagnetic conjugates were prepared by immobilizing mouse IgG recognizing target antigens onto antimouse IgG-coated magnetic beads. Conjugates targeting host antigens such as HLA-DR, CD44, CD26, β_2_-microglobulin, and Tim-4 displayed superior capture efficiency for spiked inactivated virions (grown in HUT-78 cells) or live virions in clinical plasma specimens compared to viral antigens gp120 and gp41. Cantin et al. have also demonstrated the effective capture of virions grown in HUT-78 cells targeting HLA-DR.^48^ The low capture efficiency for gp120 and gp41 could be attributed to many factors. First, gp120 and gp41 are present in limited quantities on the virion surface, whereas host-derived proteins like HLA-DR and β_2_-microglobulin are more abundantly incorporated into the viral envelope during budding.^33,34,40,49,50^ Second, the high glycosylation of gp120 forms a glycan shield, reducing accessibility for immunomagnetic conjugates.^51–53^ Furthermore, in PLHIV, circulating antibodies can bind to gp120 and gp41, forming immune complexes that mask these antigens on the virion surface and hinder conjugate binding.^54^ Lastly, gp120 and gp41 exhibit a high degree of genetic and structural variability among different HIV strains, which may limit the binding efficiency of immunoconjugates.^55^

We further evaluated capture performance under defined boundary conditions relevant to microvolume viral load testing, including conjugate amount, incubation time, and viral load. Increasing the conjugate concentration from 0.71–5.71 mg/mL resulted in a nearly 20% improvement in capture efficiency, suggesting that increasing the availability of immunomagnetic conjugates enhances binding to target antigens on HIV virions when specimen volume and target copy number are constrained. However, no significant changes in capture efficiency were observed when incubation time was varied between 15 and 60 min or across specimens with variable VLs. This consistency indicates that effective antigen recognition occurs rapidly and does not require prolonged incubation or high target abundance. This is particularly important in small-volume assays, where stochastic losses disproportionately affect analytical performance.

The anti-CD44 conjugates exhibited the highest mean capture efficiency at approximately 50% in HIV-positive plasma, aligning with prior studies reporting capture efficiencies of 26 to 70%.^33,34^ However, against inactivated virions grown in the HUT-78 cell line and spiked in buffer, the efficiency was notably lower at 24%. Interestingly, CD46 and CD43 conjugates demonstrated capture efficiencies of 29 and 37%, respectively, in HIV-positive plasma samples, despite showing no discernible capture against inactivated virions. HLA-DR immunoconjugates showed a 28% capture efficiency in HIV-positive plasma, compared to a significantly higher 62% capture efficiency against inactivated virions. Similarly, β_2_-microglobulin exhibited a capture efficiency of 14% in HIV-positive plasma versus 22% in inactivated virions. These findings suggest variable antigen expression on virions derived from HUT-78 cell lines and those circulating in PLHIV, potentially reflecting a broader pool of host cell types (e.g., macrophages and T-lymphocytes) from which virions in patients originate and acquire antigens. Previous research established that markers like CD44, HLA-DR, and β_2_-microglobulin are dual markers present on virions derived from both macrophages and T-lymphocytes. Thus, targeting these dual markers allows for effective capture of virions originating from diverse cell types.^33^

Though we did not evaluate the capture efficiency variation across infection stages (acute or chronic), our results, along with existing literature, suggest that antigen expression on virions varies depending on the cell types in which they originate during different infection stages, potentially impacting capture efficiency. Previous studies have shown that CD44-based capture efficiency remains relatively consistent between acute and chronic infection stages, while HLA-DR-based capture efficiency increases during chronic infection.^33^ Similarly, Guzzo et al. reported that antigens like integrin-α4β7 exhibit higher incorporation into virions during the acute phase but decline in the chronic phase, resulting in variable capture efficiencies.^40^ These findings underscore the dynamic nature of host antigen expression on virions, and further emphasize the importance of selecting host-derived targets that maintain consistent expression across diverse biological contexts, particularly when the goal is reproducible recovery from limited sample volumes.

We did not observe any improvement in virion capture from patient specimens when CD44 and CD46 immunoconjugates were used together compared to using either one alone. This indicates that both epitopes are likely coexpressed on the same virions and can be efficiently captured using individual conjugates, with no added benefit from their combination. However, combining immunoconjugates targeting host antigens specific to distinct cell types, such as macrophages and T-lymphocytes, from which virions originate, could potentially enhance capture efficiency and improve sensitivity for detecting VLs.

Although this study does not assess immunocapture within a fully integrated sample-to-result workflow, the observed recovery from 25-μL plasma demonstrates that immunomagnetic enrichment can increase the effective analytical input available from small specimens and has the potential to improve the practical LoD relative to direct extraction. The analytical benefit of immunomagnetic virion capture in this study is therefore evaluated relative to the extraction losses that are known to dominate sensitivity in microvolume assays, rather than relative to fully optimized, end-to-end diagnostic systems. Prior studies across multiple viral platforms have demonstrated that RNA extraction efficiency strongly governs downstream detection performance at low input volumes and copy numbers, with inefficient recovery leading to substantial signal loss and false-negative results.^56–58^ For example, Guan et al. reported that although an optimized extraction workflow (∼80% efficiency) enabled detection down to 5 copies per reaction, RNA recovery decreased to 30–50% when processing 40 μL plasma containing ∼10^3^ copies/mL.^59^ In contrast, our results demonstrate near-quantitative recovery (∼100%) from 25 μL plasma containing ∼6,000–16,000 copies/mL, establishing that immunomagnetic virion capture can preserve a substantially larger fraction of available target under low-volume conditions. While this study does not claim superior limits of detection compared with fully integrated platforms reported elsewhere,^60–62^ the observed recovery indicates that immunomagnetic enrichment can materially increase the effective analytical input available to downstream amplification assays compared with direct extraction approaches that incur disproportionate losses in the microvolume regime. We acknowledge, however, that the current data do not establish whether the observed capture efficiencies are sufficient to lower the effective LoD in a fully integrated, small-volume assay. Benchtop RT-qPCR was used here to rigorously evaluate the capture step, enabling the assessment of its efficiency, specificity, and reproducibility independent of downstream amplification biases, while defining operating conditions that can be transferred to integrated formats. In summary, this study defines the analytical behavior and operational boundaries of immunomagnetic HIV virion capture in microvolume plasma specimens. This approach demonstrated rapid capture within 15 min, detecting as few as ∼148 HIV RNA copies in 25 μL HIV-positive plasma. Notably, the capture efficiency was consistent across all tested patient samples and remained unaffected by variations in VL from 100,000 to >1,000,000 copies/mL. Host-derived markers such as CD44, HLA-DR, and β2-microglobulin were particularly effective targets, enabling selective virion recovery despite variability in antigen incorporation between cell line–derived and patient-derived virions. Rather than positioning capture efficiency as an end point, these results demonstrate how reproducible virion recovery can maintain effective analytical input in small specimens. This is a prerequisite for achieving sensitive viral load measurements when specimen volume is limited. By functioning as a plug-and-play sample-processing module, this immunomagnetic capture approach can be integrated upstream of diverse amplification and detection technologies, supporting the future development of low-volume HIV testing workflows without reliance on large plasma inputs or extensive preprocessing, and extending its analytical utility beyond the specific target and workflow evaluated here. Notably, the use of standardized, commercially available reagents and kits allows direct comparison with existing small-volume workflows, helps isolate the analytical contribution of the capture step independent of materials innovation, and enhances reproducibility and adoption by other laboratories. These attributes are central to the development of analytical methods.

## Supplementary Material



## Notes

Mouse IgG binding titration (PDF)

## #

N.P. – Department of Biomedical Engineering, The University of Texas at Austin, TX 78712, United States

## ∥

HDT Bio, 1150 Eastlake Ave E, Suite 200A, Seattle, WA 98109.
