# Supplementary material for: Immunomagnetic Sample Preparation Targeting Host- and Viral-Derived Antigens for HIV‑1 Isolation from Limited Plasma Volumes
Source: Anal Chem. 2026 Apr 29;98(18):13258–68. doi: 10.1021/acs.analchem.5c06176 (PMC13177288; doi:10.1021/acs.analchem.5c06176)
Supplement: Supplementary file 1 [file ac5c06176_si_001.pdf]

Title

Immunomagnetic Sample Preparation Targeting Host- and Viral-Derived Antigens for HIV-1 Isolation from Limited Plasma Volumes

Authors

Gaurav K. Gulati[1], Nuttada Panpradist[1†], Barry R. Lutz[1], James J. Lai[1,2*]

Affiliations

[1]Department of Bioengineering, University of Washington, Seattle, WA 98195, United States

[2]Department of Materials Science and Engineering, National Taiwan University of Science and Technology, Taipei 106335, TAIWAN

*Corresponding author. jameslai@mail.ntust.edu.tw

†Present Addresses. Department of Biomedical Engineering, The University of Texas at Austin, TX 78712, United States

Abstract (Supporting Information)

This Supporting Information provides supplementary data supporting the development of immunomagnetic conjugates for HIV-1 virion capture from small-volume plasma samples. It includes characterization of mouse IgG antibody immobilization onto anti-mouse IgG magnetic beads through binding efficiency (K_d) analysis.

Table of Contents

Figure S1. Immobilization of Alexa Fluor (AF)-labeled mouse IgG antibody onto anti-mouse IgG magnetic beads

Figure S1.

Immobilization of Alexa-Fluor (AF) labeled mouse IgG antibody onto anti-mouse IgG magnetic beads.

(A) Schematic representation illustrating the affinity binding of mouse IgG antibody with anti-mouse IgG magnetic beads. K_d represents the equilibrium dissociation constant, indicating the concentration of beads at which 50% of the mouse IgG is bound when the system is at equilibrium.

(B) Binding curve depicting the percentage of bound Alexa-Fluor (AF) labeled mouse IgG antibody to anti-mouse IgG magnetic beads. In this experiment, anti-mouse IgG magnetic beads at concentrations ranging from 0 to 2.6 mg/ml were incubated with 3.3 μg/ml of AF-labeled mouse IgG antibody within a buffer system. After a 20-minute incubation and 3 minutes of magnetic isolation, the supernatant was collected for fluorescence analysis. The percentage of bound AF-labeled mouse IgG antibody was calculated by subtracting the percentage fluorescence intensity of the unbound AF-labeled mouse IgG in the supernatant relative to a control sample lacking beads but containing AF-labeled mouse IgG, from 100.

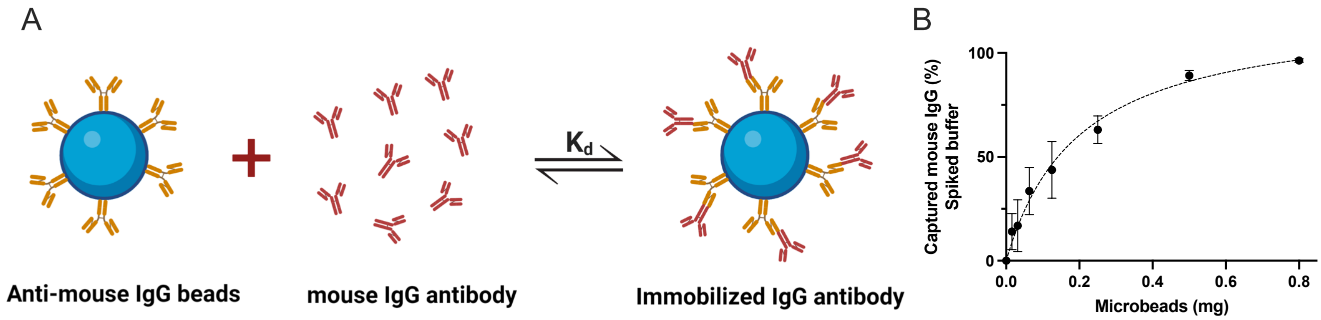
A
Kd
Anti-mouse IgG beads
mouse IgG antibody
Immobilized IgG antibody
B
Captured mouse IgG (%)
Spiked buffer
100
50
0
0.0
0.2
0.4
0.6
0.8
Microbeads (mg)
